# Baastrup’s Disease: An Often Missed Etiology for Back Pain

**DOI:** 10.7759/cureus.465

**Published:** 2016-01-22

**Authors:** Lucas R Philipp, Griffin R Baum, Jonathan A Grossberg, Faiz U Ahmad

**Affiliations:** 1 Department of Neurological Surgery, Emory University School of Medicine

**Keywords:** back pain, baastrup's disease

## Abstract

Baastrup’s disease is a relatively common disorder of the vertebral column, characterized by low back pain arising from the close approximation of adjacent posterior spinous processes and resultant degenerative changes, most commonly at L4-L5. Though fairly common, Baastrup’s disease is overwhelmingly underdiagnosed and often missed due to a lack of knowledge and/or improper diagnostic techniques, leading to frequent mistreatment. We present a case of a 56-year-old man who presented with chronic, ongoing low back pain of several years duration. His pain was relieved by flexion of the spine, and aggravated by extension. Imaging studies revealed “kissing” posterior spinous processes, consistent with a diagnosis of Baastrup’s Disease. He was treated with subcutaneous steroid injections and showed considerable clinical improvement.

## Introduction

Baastrup’s Disease (Baastrup syndrome) is a relatively common pathology affecting the vertebral column. First formally described by Christian Ingerslev Baastrup in 1933, the disorder is characterized by the close approximation and contact of the spinous processes of adjacent vertebrae in the setting of degenerative spine disease [1-2]. As a product of the radiological findings of this process, the syndrome is often referred to as the “kissing spine” syndrome. The impingement of these hypertrophied spinous processes may yield a reactive sclerosis, remodeling, and further spinal degeneration [3]. Academically, Baastrup’s Disease is a well-documented cause of low back pain, with some studies reporting an incidence as high as 81% in patients older than 80 years, though the actual prevalence is unknown [4-5]. Although it is by no means a rare disorder, Baastrup syndrome is frequently missed by clinicians due to lack of knowledge or poor imaging technique. As a result, this relatively common pathology is largely underdiagnosed and subsequently mistreated [6-7].

Here, we present a case of a 56-year-old man with a chief complaint of low back pain of several years duration. Through this case, we attempt to illustrate the characteristic features of Baastrup’s disease in its clinical presentation and key diagnostic findings. With consideration of the information presented herein, it is our hope to further educate the medical and academic community regarding this highly prevalent, commonly misdiagnosed, and erroneously treated condition. 

## Case presentation

The patient is a 56-year-old man who presented with chronic, ongoing, low back pain of several years duration. The pain was predominately axial in nature, with intermittent radiation in a craniocaudal fashion along the paraspinal regions. His pain was markedly worsened with extension of the back and improved with flexion. Pain limited his ability to extend his lumbar spine. It did not radiate to the legs, and there was no associated weakness, numbness, or bladder/bowel complaints.

His past medical history included psoriasis and hypertension, and his past surgical history included a cholecystectomy and sacroiliac iliac joint injections for arthritis.

The differential diagnosis included lumbar spondylosis, degenerative disc disease, herniated nucleus pulposus, and spinal stenosis. History and physical exam, however, provided sufficient information to effectively rule out several of these diagnoses.

On physical exam, he was neurologically intact, with no focal neurologic defects. Our patient had no leg pain or weakness, no radiation along peripheral nerve distributions, and no sensory deficits or paresthesias. There was mild to moderate tenderness in the midline in the lower back, with limited ability to extend at the spine. Baastrup’s disease became the most likely diagnosis when considering the relief of pain with flexion, and exacerbation with extension of the spine.

**Figure 1:**
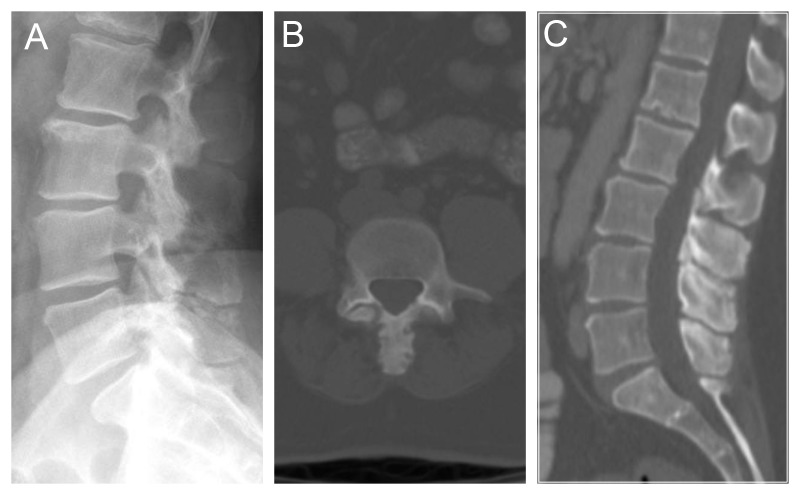
Radiographic findings on plain film and CT Mild lumbar spondylosis with prominent, hypertrophic spinous processes, contacting one another between L3 and L5 with adjacent sclerosis.

Ultimately, imaging studies confirmed our clinical suspicion. CT scan revealed mild, lumbar spondylosis with prominent and hypertrophic spinous processes that were touching each other with adjacent sclerosis (Figure 1).

**Figure 2:**
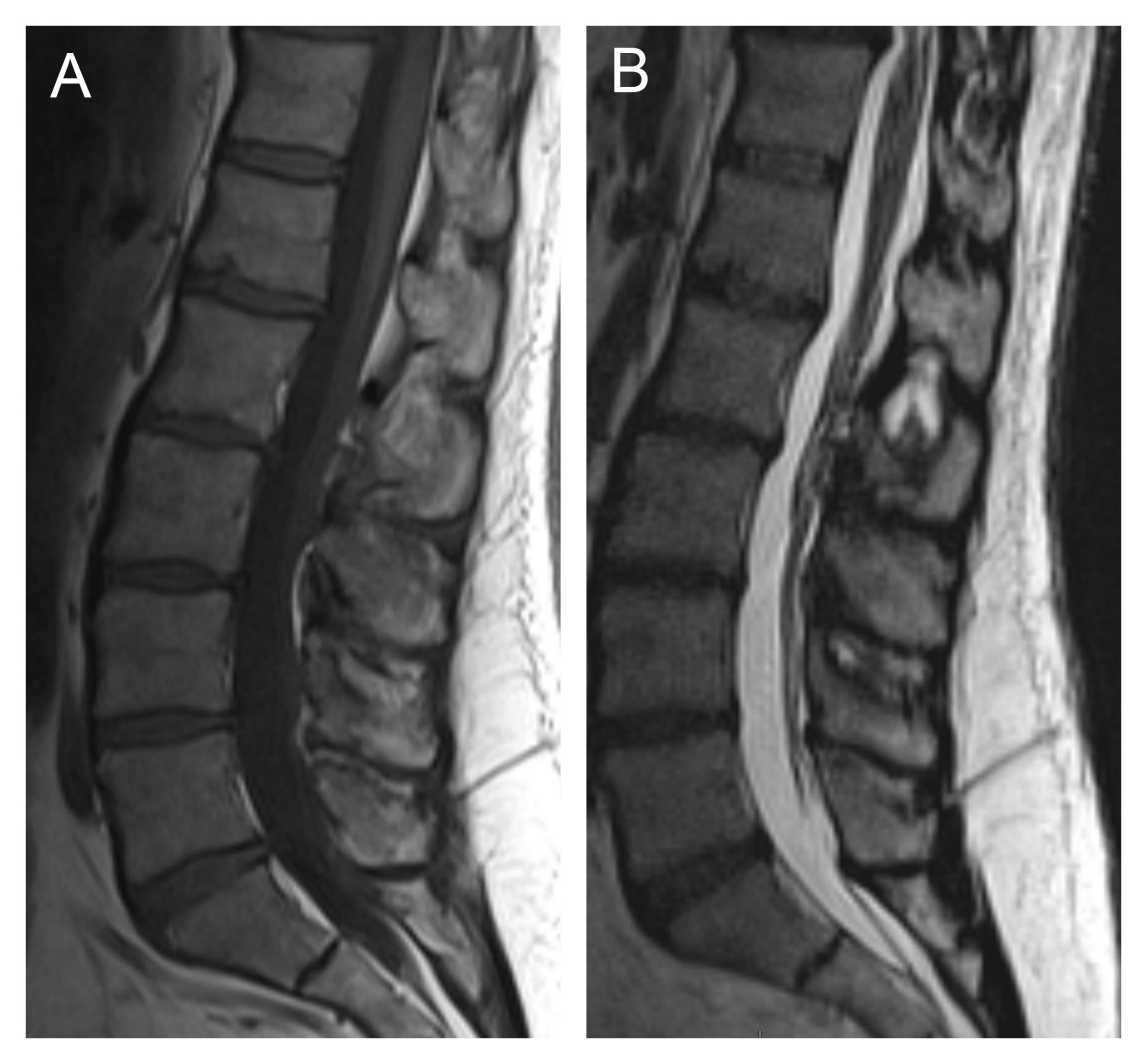
Radiographic findings on MRI T1 (A) and T2 (B) weighted imaging showing sclerotic and hypertrophic “kissing” spinous processes from L2 to L5, with mild L5-S1 facet hypertrophy

MRI corroborated these findings, with sclerotic and hypertrophic “kissing” spinous processes from L2 to L5, with mild L5-S1 facet hypertrophy (Figure 2). There was no significant spinal canal or neural foraminal stenosis. The patient was treated with steroid injections under fluoroscopic guidance with marked symptomatic relief.

## Discussion

Baastrup’s disease—“kissing spine disease”—is a relatively common disorder of the vertebral column, characterized by low back pain arising from the close approximation of adjacent, posterior spinous processes and resultant degenerative changes.

The disease most often affects the lumbar spine between L4-L5, and in the majority of cases only involves a single level [5]. Chronic contact between spinous processes may induce osseous hypertrophy and eburnation at this pathological joint. Repetitive shear force at the opposing surfaces can yield further architectural distortion and sclerosis, and contribute to the formation of interspinal adventitial bursae and cysts. Extension of this inflammation through the ligamentum flavum may also contribute to central canal stenosis [3,8].

Though it has been documented as arising idiopathically and independent of other pathology [2,9], the changes seen in Baastrup’s syndrome are typically associated with other degenerative elements including degenerative disc disease with loss of disc height, spondylolisthesis, and spondylosis [5]. As intuition may suggest, these changes are most frequently seen in elderly patients, owing to the repetitive strain and mechanical pressure produced by excessive lordosis; persistent strain on the interspinous ligament may consequentially yield further degeneration and collapse [2,4-5]. Additionally, as a product of repetitive spinal flexion and extension, Baastrup’s syndrome has been seen clinically in 6.3% of college athletes, most notably gymnasts [10]. For this reason, it is important to consider Baastrup’s syndrome in vulnerable populations who lie outside of the anticipated age demographic for degenerative spine disease.

Other documented etiologies include poor posture and traumatic injury. Any condition which contributes to excessive lordosis may also produce Baastrup’s syndrome including kyphoscoliosis, tuberculous spondylitis, stiff thoracolumbar transition, obesity, and bilateral forms of congenital hip dislocation [2,5,11].

The initial clinical presentation of the disorder is most often characterized by low back pain, and although rarely, the cervical spine may be involved [2,12]. The pain is described as midline, in the lumbar region, and with radiation along the spine but not laterally. Symptoms are relieved with spinal flexion and aggravated by extension, and the pain can be elicited clinically by palpation of the affected interspinous space. In the setting of secondary central canal disease, leg pain and weakness have been described with standing or walking in a fashion consistent with neurogenic claudication [2,8,13]. Clinical maneuvers which passively or actively extend the lumbar spine are useful in reproducing symptoms.

Diagnosis is dependent upon characteristic findings on imaging studies. The ‘kissing’ of closely approximated spinous processes can often be seen on lateral plain film X-rays, sometimes with visible sclerosis of the articulating surfaces [3]. Computed Tomography is suitable for visualizing these bony changes and may also show generalized degenerative changes in greater detail; however, neither CT nor plain films are suitable for demonstrating pathological changes in the soft tissues of the spine. Frequently, Baastrup’s disease is missed due to lack of knowledge and overexposure of the spinous processes [7].

MRI is the most sensitive imaging modality for detecting Baastrup’s disease and may do so much earlier in the disease course. It has been noted that interspinous bursitis may precede the more pronounced osseous changes of the spinous processes, which MRI is the best suited for detecting [3]. The bursae appear as bright, high-intensity areas on T2-weighted MRI, between posterior spinous processes. Additionally, MRI may show reactive sclerosis and hypertrophy of the spinous processes--which may have flattened and enlarged articulating surfaces, may show associated edema at the level of the interspinous ligament, and provides insight into the degree to which the posterior thecal sac is compressed [11,14].

Treatment of Baastrup’s syndrome is an ongoing topic of debate. Traditionally, surgical techniques have been employed including excision of the bursae and osteotomy to shorten the offending spinous processes [2].  Other studies suggest that these osteotomy-only techniques are ineffective in relieving symptoms [15]. The use of interlaminar stabilization devices has not been investigated in patients with Baastrup's disease, but could be paired with an osteotomy procedure. Alternatively, percutaneous injections of long-acting corticosteroids and sometimes local anesthetics have been used to treat inflammation and pain [16-18]. Results have shown significant improvement in pain scores at over one year following treatment [18]. Physical therapy also plays an important role in the long-term management of Baastrup’s syndrome and focuses on reducing interspinous strain and lordosis.

Baastrup’s disease is underdiagnosed and often missed, resulting in incorrect treatment. Some studies have suggested that Baastrup’s disease may not be mutually exclusive from other causes of degenerative spine disease, and multiple pathologies may coexist simultaneously [7,11]. Furthermore, degeneration of one anatomical component of the spine may induce degeneration of other spinal elements [19]. Although the most common primary pathologies responsible for low back pain are intervertebral discs and facet joints, it is important to consider spinal elements lying outside of the vertebral bodies when generating a differential diagnosis for axial back pain.

## Conclusions

Careful clinical evaluation of patients and critical analysis of imaging studies are necessary for the correct diagnosis and subsequent treatment of all causes of low back pain. Baastrup’s disease is characterized by “kissing” posterior spinous processes of the spine, resulting in pain with spinal extension, morphological changes of the vertebrae, and further spinal degeneration as best seen on MRI. Though this condition is presumed to be a relatively common etiology for low back pain, it is overwhelmingly underdiagnosed and often missed due to a lack of knowledge and/or improper diagnostic techniques. As such, these individuals are frequently mistreated and may undergo unnecessary procedures which are ineffective in addressing the underlying etiology of their pain. Clinically, the pain resulting from Baastrup’s disease is relieved by flexion of the spine and aggravated by extension of the spine or palpation. Treatment remains a subject of debate, though percutaneous corticosteroid injections have been shown to be beneficial for symptom relief. 
